# Nano-LC-MS/MS for Quantification of Lyso-Gb3 and Its Analogues Reveals a Useful Biomarker for Fabry Disease

**DOI:** 10.1371/journal.pone.0127048

**Published:** 2015-05-12

**Authors:** Hideaki Sueoka, Junji Ichihara, Takahiro Tsukimura, Tadayasu Togawa, Hitoshi Sakuraba

**Affiliations:** 1 Genomic Science Laboratories, Sumitomo Dainippon Pharma Co., Ltd., 3-1-98 Kasugade-naka, Konohana-ku, Osaka 554–0022, Japan; 2 Drug Development Research Laboratories, Sumitomo Dainippon Pharma Co., Ltd., 33–94 Enokicho, Suita, Osaka 564–0053, Japan; 3 Department of Functional Bioanalysis, Meiji Pharmaceutical University, 2-522-1 Noshio, Kiyose, Tokyo 204–8588, Japan; 4 Department of Clinical Genetics, Meiji Pharmaceutical University, 2-522-1 Noshio, Kiyose, Tokyo 204–8588, Japan; University of Würzburg, GERMANY

## Abstract

Biomarkers useful for diagnosis and evaluation of treatment for patients with Fabry disease are urgently needed. Recently, plasma globotriaosylsphingosine (lyso-Gb3) and lyso-Gb3-related analogues have attracted attention as promising biomarkers of Fabry disease. However, the plasma concentrations of lyso-Gb3 and its analogues are extremely low or below the detection limits in some Fabry patients as well as in healthy subjects. In this paper, we introduce the novel application of a nano-liquid chromatography-tandem mass spectrometry (nano-LC-MS/MS) system to the measurement of lyso-Gb3 and its analogues in plasma. Nano-LC-MS/MS requires smaller amounts of samples and is more sensitive than conventional techniques. Using this method, we measured the plasma concentrations of lyso-Gb3 and its analogues in 40 healthy subjects, 5 functional variants (males with E66Q), and various Fabry patients (9 classic Fabry males/9 mutations; 7 later-onset Fabry males/5 mutations; and 10 Fabry females/9 mutations). The results revealed that the mean lyso-Gb3 and lyso-Gb3(-2) concentrations in all the Fabry patient subgroups were statistically higher, especially in the classic Fabry males, than those in the functional variants and healthy subjects. The plasma concentrations of lyso-Gb3 and its analogues in healthy subjects, functional variants, and some Fabry patients with specific mutations (R112H and M296I) that cannot be established by conventional techniques were successfully determined by means of nano-LC-MS/MS. The lyso-Gb3 and lyso-Gb3(-2) concentrations in male patients with these mutations were lower than those in most Fabry patients having other mutations, but higher than those in the functional variants and healthy subjects. This new method is expected to be useful for sensitive determination of the plasma concentrations of lyso-Gb3 and its analogues. This study also revealed that not only lyso-Gb3 but also lyso-Gb3(-2) in plasma is a useful biomarker for the diagnosis of Fabry disease.

## Introduction

Fabry disease (OMIM No. 301500) is an X-linked lysosomal storage disorder caused by a deficiency of an enzyme, α-galactosidase A (GLA, EC 3.2.1.22). The GLA activity deficiency results in progressive accumulation of glycosphingolipids, predominantly globotriaosylceramide (Gb3), in organ tissues and body fluids [1]. Fabry disease exhibits clinical phenotypes ranging from the early-onset “classic form” to the “later-onset form”. Affected males with the classic form exhibit little or no GLA activity, and pain in the peripheral extremities, hypohidrosis, angiokeratomas, and corneal opacities in childhood or adolescence, and then develop kidney, cardiac and cerebrovascular disorders in adulthood. Patients with the later-onset form usually have low residual GLA activity, and show milder clinical manifestations limited to the heart and/or kidneys [2]. The disease expression in Fabry females depends on random X-chromosomal inactivation and the clinical phenotypes are more heterogeneous than in males [3].

Since enzyme replacement therapy (ERT) involving recombinant human GLAs is available for the treatment of Fabry disease [4,5], biomarkers of the disease have become more and more important for diagnosis, monitoring of disease progression, and assessment of therapeutic efficacy. The plasma Gb3 level has been measured for the above purposes, but recent investigations revealed that the plasma Gb3 levels in later-onset Fabry males and Fabry females were not necessarily higher than those in healthy subjects. Thus, Gb3 is not necessarily an ideal biomarker for diagnosis or the response to treatment [6,7]. Plasma globotriaosylsphingosine (lyso-Gb3), a deacylated derivative of Gb3, has been reported to be a promising biomarker for the diagnosis of Fabry patients, particularly later-onset and female patients [7–11]. Some early papers reported that the plasma lyso-Gb3 concentration in later-onset patients harboring a specific mutation, i.e., R112H or M296I, was below or near the detection limit of 2–3 nM [7–9,12]. In these studies, high-performance liquid chromatography (HPLC) methods involving o-phthaldialdehyde (OPA)-derivatization and fluorescence detection, which are not highly sensitive as to this substrate, were used for determination of lyso-Gb3. Methods involving liquid chromatography-mass spectrometry (LC-MS) have also been reported as more sensitive means of quantifying lyso-Gb3 [11,13–17]. However, there has been no report on LC-MS determination of the plasma lyso-Gb3 concentration in Fabry patients with R112H or M296I. Recently, lyso-Gb3-related analogues were identified as novel biomarkers of Fabry disease on metabolomics analysis of urine or plasma from Fabry patients [16–19]. The plasma concentrations of these analogues are lower than that of lyso-Gb3, and are not measurable in some Fabry patients and healthy subjects.

To determine trace amounts of lyso-Gb3 or its analogues in human plasma, we have applied a nano-liquid chromatography-MS/MS system (nano-LC-MS/MS). A nano-LC system was reported for the first time in 1988 [20], and is often used in combination with a mass spectrometer for determining trace amounts of metabolites in biological samples. In general, nano-LC-MS/MS requires smaller amounts of samples and is more sensitive than conventional LC-MS/MS systems. Therefore, nano-LC-MS/MS has been applied in many fields (proteomics, lipidomics, etc.) [21–23]. As a mass spectrometer, we select a new generation benchtop Orbitrap instrument (Q-Exactive hybrid quadrupole-Orbitrap mass spectrometer), which combines high-performance quadrupole precursor selection with high resolution and accurate-mass (HR/AM) Orbitrap detection. The Q-Exactive provides MS/MS spectra of analytes passing through the quadrupole, by which target analytes can be identified. In addition, by choosing a very narrow window of fragment ion masses for quantification, more contaminants having mass-to-charge ratios (m/z) close to those of targets can be eliminated (targeted MS/MS [high resolution product scan, HRPS] analysis) compared to in the cases of other mass spectrometers. Due to these characteristics, analytes in biological samples can be quantified more accurately [24].

In this study, using the new nano-LC-MS/MS system, we determined the plasma concentrations of lyso-Gb3 and its analogues in Fabry patients with various phenotypes, functional variants (males with E66Q), and healthy subjects.

## Materials and Methods

### Ethics Statement

This study was performed according to the ethical guidelines of the 1975 Declaration of Helsinki. Written informed consent was obtained from all the patients examined. As to the children enrolled in this study, written informed consent was obtained from their guardians on behalf of them. The study was approved by the Ethical Committees of Sumitomo Dainippon Pharma Co., Ltd., and Meiji Pharmaceutical University.

### Reagents

Lyso-Gb3 was purchased from Matreya, LLC (Pleasant Gap, PA, USA). Stable-isotope labeled lyso-Gb3 (lyso-Gb3-IS), as an internal standard (IS), was synthesized according to a previous report [25] at Nard Institute, Ltd. (Kobe, Japan). Two stable-isotope labeled chemicals were used for lyso-Gb3-IS synthesis: L-Serine-1-^13^C (isotopical purity, 99%; Cambridge Isotope Labolatories, Inc., Tewksbury, MA, USA), and palmitoic acid-CD_3_ (isotopical purity, 99%; ISOTEC, Inc., Miamisburg, OH, USA). Lyso-Gb3-IS has one ^13^C and 3 deuteriums. For sample preparation and nano-LC-MS/MS analysis, ammonia water (NH_4_OH) was purchased from Sigma-Aldrich Co., LLC. (St. Louis, MO, USA). Phosphoric acid (H_3_PO_4_), LC-MS grade methanol (MeOH), and acetonitrile (ACN) were purchased from Kanto Chemical Co., Inc. (Tokyo, Japan), and LC-MS grade formic acid (FA) from Wako Pure Chemical Industries, Ltd. (Osaka, Japan). Charcoal-treated plasma was prepared in our laboratory.

### Subjects and Samples

Plasma samples were obtained from nine male subjects with classic Fabry disease (age range, 11–58 years; median age, 40 years), seven male subjects with later-onset Fabry disease (age range, 14–62 years; median age, 54 years), and ten heterozygous females with Fabry disease (age range, 8–70 years; median age, 46 years). All plasma samples from Fabry patients were collected before enzyme replacement therapy. The classification of the Fabry patients was performed according to the clinical manifestations, family history and results of gene analysis. Fabry disease mutations were classified according to the Fabry database (http://fabry-database.org/). To determine a trace amount of lyso-Gb3, Fabry patients having the R112H or M296I mutation, in whom the lyso-Gb3 concentration is expected to be low, were included in the data set. Of the seven later-onset Fabry males, two harbored the R112H mutation and two the M296I one. Of the ten heterozygous Fabry females, one harbored the R112H mutation and two the M296I one. The ages, genotypes and clinical manifestations of the Fabry patients are summarized in Table 1. For comparison, plasma samples from five functional variants (males with E66Q) (age range, 45–78 years; median age, 71 years) and 40 healthy subjects were used. The plasma samples from healthy subjects were purchased from Tissue Solutions, Ltd. (Glasgow, UK); 20 males (age range, 26–71 years; median age, 41 years) and 20 females (age range, 19–55 years; median age, 36 years).

**Table 1:** Genotypes, plasma lyso-Gb3 concentrations, and clinical manifestations in the patients with Fabry disease.

Group	Age (yr)	Genotype	Lyso-Gb3 (nM) /nano-LC	Lyso-Gb3 (nM) /HPLC	Clinical manifestations (P/A/HH/CO)	Clinical manifestations (RD/HD/CV)
Classic Fabry male 1	11	c.85delG	43	36*	-/-/+/-	-/-/-
2	47	p.G43V	1.8x10^2^	1.1x10^2^	-/-/+/N	-/+/-
3	58	p.R112C	1.1x10^2^	53	+/+/+/-	+/+/+
4	38	c.370delG	1.9x10^2^	1.3x10^2^	N/N/N/N	N/N/N
5	23	p.G147E	1.4x10^2^	91*	N/N/N/N	N/N/N
6	35	c.718_719delAA	1.1x10^2^	67	+/-/+/-	+/-/+
7	40	p.S345X	1.8x10^2^	92	+/+/+/+	+/+/-
8	46	p.E66Q/p.I354K	1.1x10^2^	71*	-/-/-/+	+/+/+
9	42	p.W399X	1.6x10^2^	34	-/N/N/N	+/+/+
Mean±SD	38±14		1.4x10^2^±47	76±33		
Later-onset Fabry male 1	45	p.R112H	1.6	<2*	-/-/-/-	+/-/-
2	14	p.R112H	4.1	4.9*	-/-/-/N	+/-/-
3	31	p.P210L	9.6	11	-/-/-/N	+/+/-
4	60	p.N215S	5.8	4.2*	-/-/-/-	+/+/-
5	61	p.M296I	2.3	<2*	-/-/-/N	+/+/-
6	54	p.M296I	3.2	<2*	N/N/N/N	-/-/-
7	62	p.R301Q	46	15*	N/-/-/N	-/+/-
Mean±SD	47±18		10±16	4.4±5.8		
Fabry female 1	44	c.85delG/WT	7.8	5	-/N/-/-	-/+/-
2	34	p.M42V/WT	13	7*	+/-/-/+	+/+/-
3	49	p.R112H/WT	0.75	<2*	N/N/N/N	N/N/N
4	66	p.G147E/WT	25	8	N/N/N/N	N/N/N
5	59	p.R227X/WT	26	12*	+/-/N/+	-/+/+
6	28	p.W245X/WT	6.2	6*	+/-/-/+	-/-/-
7	33	p.M296I/WT	0.91	<2*	-/-/-/-	-/-/-
8	70	p.M296I/WT	0.85	<2*	+/-/+/N	+/-/-
9	47	c.1033_1034delTC/WT	23	16	-/+/-/+	+/+/-
10	8	p.Y365X/WT	1.5 x10^2^	1.4x10^2^	+/-/+/N	N/N/N
Mean±SD	44±19		25±44	19±41		
Functional variants (n = 5)	65±13	p.E66Q	0.55±0.20	<2		
Healthy subjects (n = 40)	40±13		0.37±0.11	<2		

P: Pain in peripheral extremities, A: Angiokeratomas, HH: Hypohidrosis, CO: Corneal opacities, RD: Renal disease, HD: Heart disease, and CV: Cerebrovascular disease. N: Not available. WT: Wild type

*Ref. 30.

### Sample Preparation

Extraction of lyso-Gb3 and its analogues from plasma was performed by a modification of the method described in a previous report [15]. As a standard solution, charcoal-treated plasma was spiked with lyso-Gb3 at 0 to 250 nM. As an IS solution, lyso-Gb3-IS was diluted in 1% H_3_PO_4_/MeOH to 2.5 nM. Twenty μL aliquots of the standard solution or plasma samples from the patients, functional variants and healthy subjects were each added to 80 μL of the IS solution, followed by mixing and centrifugation for protein precipitation to remove insoluble protein. After centrifugation, an 80 μL aliquot of each supernatant was diluted in 920 μL of 1% H_3_PO_4_/MeOH, followed by transfer to an OASIS MCX cartridge (30mg, 60μm; Waters Corp., Milford, MA, USA) preconditioned with 1200 μL of MeOH and 1200 μL of 2% H_3_PO_4_. The cartridge was washed with 1200 μL of 2% FA, then with 1200 μL of 0.2% FA/MeOH, and finally with 1200 μL of 2% NH_4_OH (28%[v/v])/50% MeOH. In the case of extraction of lyso-Gb3-related analogues, the final washing step was excluded. Lyso-Gb3, its analogues and lyso-Gb3-IS were eluted into glass tubes with 1200 μL of 2% NH_4_OH (28%[v/v])/MeOH, followed by drying in an evaporator. Each residue was reconstituted in 30–40 μL of 0.1% FA/50% ACN. After sonication and centrifugation, the reconstituted sample was injected into the nano-LC MS/MS system.

### Instruments and Quantification

As a nano-LC system, an Ultimate 3000RSLCnano (Thermo Fisher Scientific, Inc., Waltham, MA, USA) and a PAL HTS XT-CTC autosampler (CTC Analytics AG, Zwingen, Switzerland) were used. The samples were injected via a 1 μL nanoViper Loop (Thermo Fisher Scientific). A Zorbax 300SB-C18 nano column (150 mm x 0.1mm, 3 μm particles; Agilent Technology, Inc., Santa Clara, CA, USA) was used for chromatographic separation of lyso-Gb3 and its analogues. Solvent A comprised 0.2% FA/5% ACN and Solvent B 0.2% FA/ACN. A mobile-phase gradient was produced during a 25 min run: 0 min, 1% B; 10 min, 99% B; 16 min, 99% B; 16.1 min, 1% B; and 25 min, 1% B. The flow rate was 0.5 μL/min. A Q-Exactive mass spectrometer (Thermo Fisher Scientific) was used for detection of lyso-Gb3 and its analogues. Instrument calibration was performed before each analysis. The targeted MS/MS analysis (HRPS) mode was selected for quantification of lyso-Gb3 and its analogues. The isolation window for the first quadrupole was 2.0 m/z. The Orbitrap spectrometer was operated at 17,500 full width at half maximum (FWHM). The AGC target value was set to 1E5, with a maximum injection time of 100 ms. The collision energy value was 25% for all the compounds of interest. The target masses were m/z 786.4482 for lyso-Gb3 and m/z 790.470 for lyso-Gb3-IS. In the case of measurement of lyso-Gb3 analogues, m/z 758.417 for lyso-Gb3(-28), m/z 774.412 for lyso-Gb3(-12), m/z 784.433 for lyso-Gb3(-2), m/z 800.427 for lyso-Gb3(+14), m/z 802.443 for lyso-Gb3(+16), m/z 804.459 for lyso-Gb3(+18), m/z 820.454 for lyso-Gb3(+34), and m/z 836.449 for lyso-Gb3(+50) were used for the targeted MS/MS analysis method. The calculation were performed with Quan Browser software (Thermo Fisher Scientific). Highest intensive fragment ions were selected for quantification, i.e., m/z 282.278 for lyso-Gb3, m/z 286.301 for lyso-Gb3-IS, m/z 254.248 for lyso-Gb3(-28), m/z 252.232 for lyso-Gb3(-12), m/z 280.263 for lyso-Gb3(-2), m/z 278.248 for lyso-Gb3(+14), m/z 280.263 for lyso-Gb3(+16), m/z 318.300 for lyso-Gb3(+18), m/z 334.295 for lyso-Gb3(+34), and m/z 350.290 for lyso-Gb3(+50). Extracted ion chromatograms of the target analytes were constructed with an optimized mass extraction window (MEW) of 10 ppm. The concentration of lyso-Gb3 was calculated from a calibration curve. The concentrations of lyso-Gb3 analogues were evaluated as to the peak area ratio to lyso-Gb3-IS, because lyso-Gb3 analogue standards were not available.

### Method Validation

#### Lyso-Gb3

A set of standard samples of six concentrations, ranging from 0.08 nM to 250 nM, was prepared for each concentration point of the calibration curve. The calibration curve was obtained using peak area ratios (lyso-Gb3/lyso-Gb3-IS) calculated from chromatograms of the samples. The regression equation and correlation coefficient (r^2^) were calculated from the standard curve. These operations were repeated three times on different days to evaluate the linearity and quantification ranges. Intra-day assaying was performed with four concentrations of samples for quality checking (QC) (QCLL = 0.08 nM, QCL = 0.4 nM, QCM = 10 nM, and QCH = 200 nM) in quintuplicate (n = 5) to evaluate the precision and accuracy within a day. Inter-day assaying was also performed with four concentrations of samples (QCL, QCM, QCH and QCLL) in quintuplicate (n = 5), and repeated five times on different days to evaluate the precision and accuracy. The recovery of lyso-Gb3 was determined for two samples at three concentrations (QCL, QCM, and QCH). Twenty μL aliquots of lyso-Gb3 spiked (recovered sample) and un-spiked (control sample) plasma were each added to 80 μL of 1% H_3_PO_4_/MeOH. After purification with an MCX cartridge, the recovered samples were reconstituted in 20 μL of 0.1% FA/50% ACN at 10 nM lyso-Gb3-IS, the control samples being reconstituted in 20 μL of 0.1% FA/50% ACN at 10 nM lyso-Gb3-IS with 0.4 nM (corresponding to QCL), 10 nM (corresponding to QCM), or 200 nM (corresponding to QCH) of lyso-Gb3. The recovery was calculated from the peak area ratios (lyso-Gb3/lyso-Gb3-IS) for two samples (recovered and control samples). The recovery of lyso-Gb3-IS was also evaluated. The matrix effect of lyso-Gb3 was evaluated by the method reported previously [17]. For assessment of the matrix effect, ten μL aliquots of pooled plasma from classic Fabry males were each combined with 10 μL of each of the following plasma samples: (1) charcoal treated plasma for the calibration curve; (2) non-treated plasma; (3) plasma from a healthy 24-year-old woman; (4) plasma from a healthy 54-year-old woman; (5) plasma from a healthy 32-year-old man; and (6) plasma from a healthy 58-year-old man. All six samples were prepared and analyzed to examine the matrix effect. Stability testing of lyso-Gb3 was also performed. Pooled plasma samples from classic Fabry males were each aliquoted and analyzed after 3 h and 6 h at room temperature (22°C), 3 h and 6h at 4°C, and 2 weeks at -20°C, and the effect of three freeze-thaw cycles was also examined. The reconstituted samples after MCX purification left in the autosampler (10°C) for 30 h were also evaluated.

#### Lyso-Gb3-related Analogues

Lyso-Gb3 analogue standards were not available. Therefore, the precision for lyso-Gb3 analogues was evaluated using the peak area ratio (each lyso-Gb3-analogue/ lyso-Gb3-IS) with high (pooled plasma from classic Fabry males), middle (mixed plasma from pooled classic Fabry males and healthy subjects [1:20]), and low [1:400] concentrations in quintuplicate (n = 5). The matrix effects of lyso-Gb3 analogues were evaluated by the method described above. The stability of two analogues, lyso-Gb3 (-12) and (+14), in plasma was investigated, since that of the other six analogues had been investigated in the previous study [17]. The stability test was performed under the same conditions as that for lyso-Gb3.

### Measurement of Lyso-Gb3 by HPLC

Plasma lyso-Gb3 was also measured by means of HPLC, followed by fluorescence detection, as described previously [7,10].

### Statistical Analysis

Data are expressed as means ± standard deviation (SD). The plasma concentrations of lyso-Gb3 and its analogues in the classic Fabry males, later-onset Fabry males, Fabry females, functional variants and healthy subjects were log transformed to improve normality in statistical analysis. The differences among these groups were assessed by means of the Tukey multiple-comparison test. The statistical significance test was performed with Stat Preclinica SAS 9.2 (Takumi Information Technology Inc., Tokyo, Japan).

## Results

### Method Validation

#### Lyso-Gb3

The quantification of lyso-Gb3 was evaluated using charcoal-treated plasma. The linearity of the calibration curve for lyso-Gb3 was evaluated by repeating the same experiments three times on different days. The correlation coefficients (r^2^) calculated from calibration curves were all more than 0.994. The intra-day CVs (%) for precision and accuracy were 3.7% and 9.1%, respectively, at QCLL, and less than 3.0% and 9.7%, respectively, at the other QCs (Table 2 and S1 Table). The inter-day CVs (%) for precision and accuracy were 18.4% and 3.1%, respectively, at QCLL, and less than 5.3% and 13.0%, respectively, at the other QCs (Table 2 and S2 Table). The limit of detection (LOD) and limit of quantification (LOQ) were 0.01 nM and 0.03 nM, respectively, LOD and LOQ being defined as 3 and 10 times the SD of the lyso-Gb3 response (area lyso-Gb3/area lyso-Gb3-IS), calculated from five QCLLs (0.08 nM), divided by the slope of the calibration curve. Although the recovery of lyso-Gb3 was around 50% for QC samples, there was no difference for any of the QCs (S3 Table). The coefficient value of variation of the recovery for the internal standard (lyso-Gb3-IS) was the same as those for QCs. The matrix effect of lyso-Gb3 was evaluated by mixing 10 μL of pooled plasma from classic Fabry males with 10 μL of six different plasma samples from healthy subjects, including charcoal-treated plasma, for the calibration curve. The measured concentrations of lyso-Gb3 provided a CV of 4.2%, indicating a very low matrix effect. The stability test showed that lyso-Gb3 was stable in plasma after three freeze/thaw cycles, and at least for 6 h at both room temperature and 4°C, and 2 weeks at -20°C. The reconstituted solution left for 30 h in the autosampler was also stable. All CVs of the stability test were under 1.9%. These results showed that nano-LC-MS/MS for quantification of plasma lyso-Gb3 was accurate, reproducible, and highly sensitive.

**Table 2:** Intra-day and inter-day precision and accuracy for controlled plasma samples spiked with lower limit (QCLL, 0.08nM), low (QCL, 0.4nM), medium (QCM, 10nM), and high (QCH, 200nM) concentrations of lyso-Gb3.

Assay	QCLL (0.08 nM)	QCL (0.4 nM)	QCM (10 nM)	QCH (200 nM)
Intra-day Precision (% CV), n = 5	3.7	3.0	2.4	1.6
Intra-day Accuracy (% Bias), n = 5	9.1	-9.7	-9.5	2.0
Inter-day Precision (% CV), n = 5	18.4	4.7	5.3	2.9
Inter-day Accuracy (% Bias), n = 5	-3.1	-13	-10.5	2.1

#### Lyso-Gb3-related analogues

**Fig 1:**
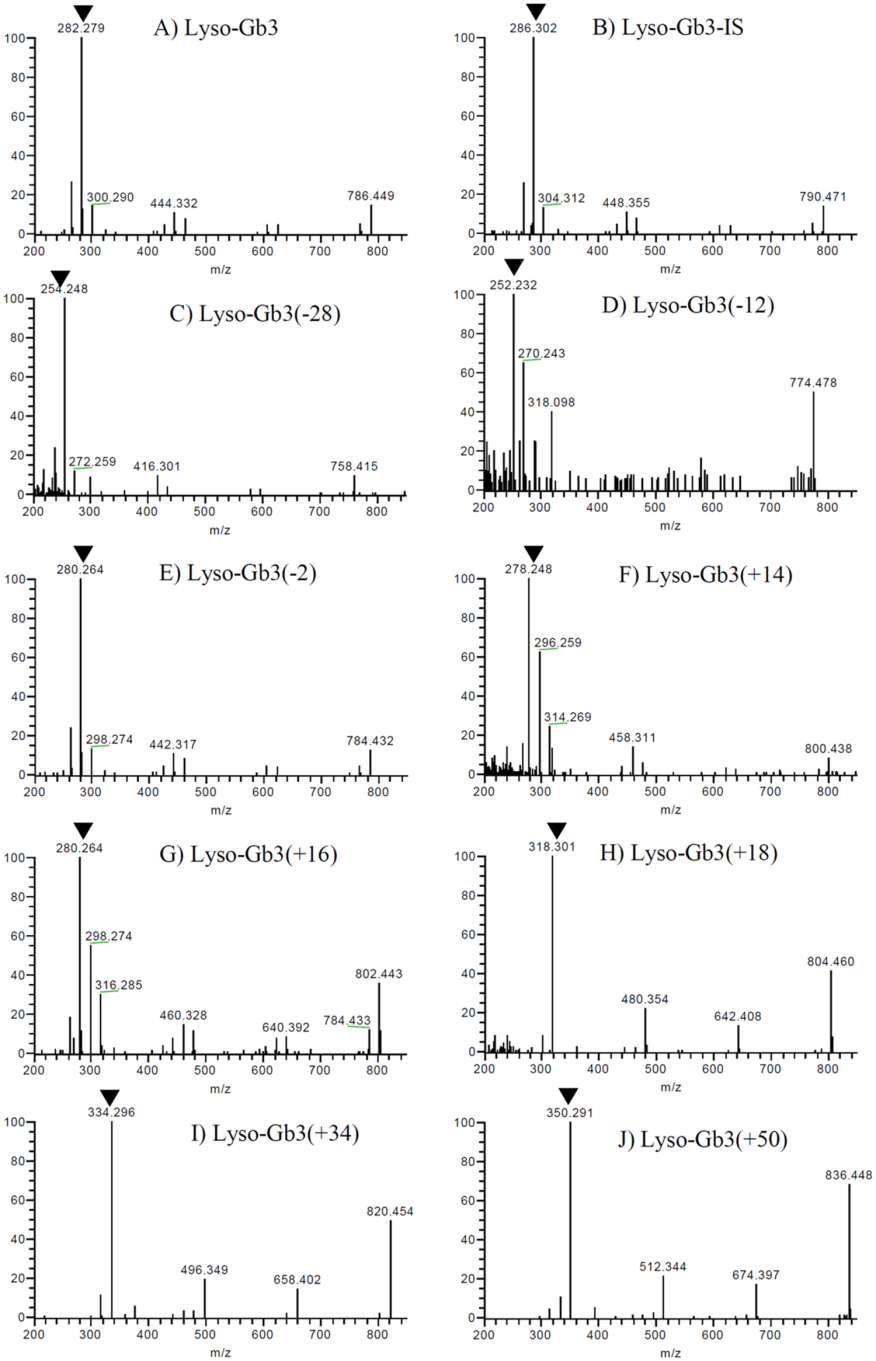
MS/MS spectra of lyso-Gb3, lyso-Gb3-IS and lyso-Gb3 analogues acquired from a classical Fabry patient with a Q-Exactive. (A) Lyso-Gb3, (B) Lyso-Gb3-IS, (C) Lyso-Gb3(-28), (D) Lyso-Gb3(-12), (E) Lyso-Gb3(-2), (F) Lyso-Gb3(+14), (G) Lyso-Gb3(+16), (H) Lyso-Gb3(+18), (I) Lyso-Gb3(+34), and (J) Lyso-Gb3(+50). Triangles (▼) indicate the target fragments used for quantification.

Various lyso-Gb3-related analogues have been discovered in the plasma and urine of Fabry patients through excellent metabolomics studies [16–19]. In our study, eight lyso-Gb3 analogues were detected in plasma samples from Fabry patients (Fig 1 and S1 Fig). The quantification of lyso-Gb3 analogues was evaluated using plasma from Fabry patients since lyso-Gb3 analogue standards were not available. The peak area ratios of eight lyso-Gb3 analogues as to lyso-Gb3-IS for the plasma from the classic Fabry males (high conc., n = 5), that from the classic Fabry males 1/20 diluted in the healthy subject plasma (middle conc., n = 5), and that from the classic Fabry males 1/400 diluted in the healthy subject plasma (low conc., n = 5) were evaluated. This assay system provided precision with a CV of 14.4% or less for quantifying lyso-Gb3(-2), lyso-Gb3(+18), and lyso-Gb3(+34) at all concentrations, lyso-Gb3(-28), lyso-Gb3(+16), and lyso-Gb3(+50) at the middle and high concentrations, and lyso-Gb3(-12) and lyso-Gb3(+14) at the high concentrations (S4 Table). The evaluation of the matrix effect of lyso-Gb3 analogues revealed low CVs of 12.8% (lyso-Gb3(-28)), 16.6% (lyso-Gb3(-12)), 12.5% (lyso-Gb3(-2)), 13.6% (lyso-Gb3(+14)), 11.8% (lyso-Gb3(+16)), 5.6% (lyso-Gb3(+18)), 9.2% (lyso-Gb3(+34)), and 15.4% (lyso-Gb3(+50)), indicating a low matrix effect. The stability test showed that lyso-Gb3(-12) and (+14) were stable in plasma after three freeze/thaw cycles for 2 weeks at -20°C, and in the reconstituted solution for 30 hours in the autosampler. The analogues were stable in plasma for 3 hours at 4°C, but not at room temperature or for 6 hours at 4°C (S5 Table). Therefore, we used clinical samples that had been stored in a deep freezer until use for the measurement of plasma lyso-Gb3 and its analogues.

### Plasma concentrations of lyso-Gb3 in Fabry patients, functional variants and healthy subjects

We measured the lyso-Gb3 concentrations in plasma samples from various Fabry patients, functional variants (males with E66Q), and healthy subjects (Table 1). The mean plasma concentrations of lyso-Gb3 were 1.4x10^2^±47 nM for the classic Fabry males (n = 9), 10±16 nM for the later-onset Fabry males (n = 7), 25±44 nM for the Fabry females (n = 10), 0.55±0.20 nM for the functional variants (n = 5), and 0.37±0.11 nM for the healthy subjects (n = 40). A statistically significant difference was found in the mean values for lyso-Gb3 between the classic Fabry males and the healthy subjects (p< 0.001). The mean lyso-Gb3 concentrations in the later-onset Fabry males and the Fabry females were significantly lower than that in the classic Fabry males (p<0.001 and p<0.001, respectively), but significantly higher than those in the functional variants and the healthy subjects (all, p<0.001). The mean lyso-Gb3 concentration in the functional variants was statistically lower than those in all the Fabry subgroups (p< 0.001), although there was no difference between the functional variant group and the healthy subject one (p> 0.05).

**Fig 2:**
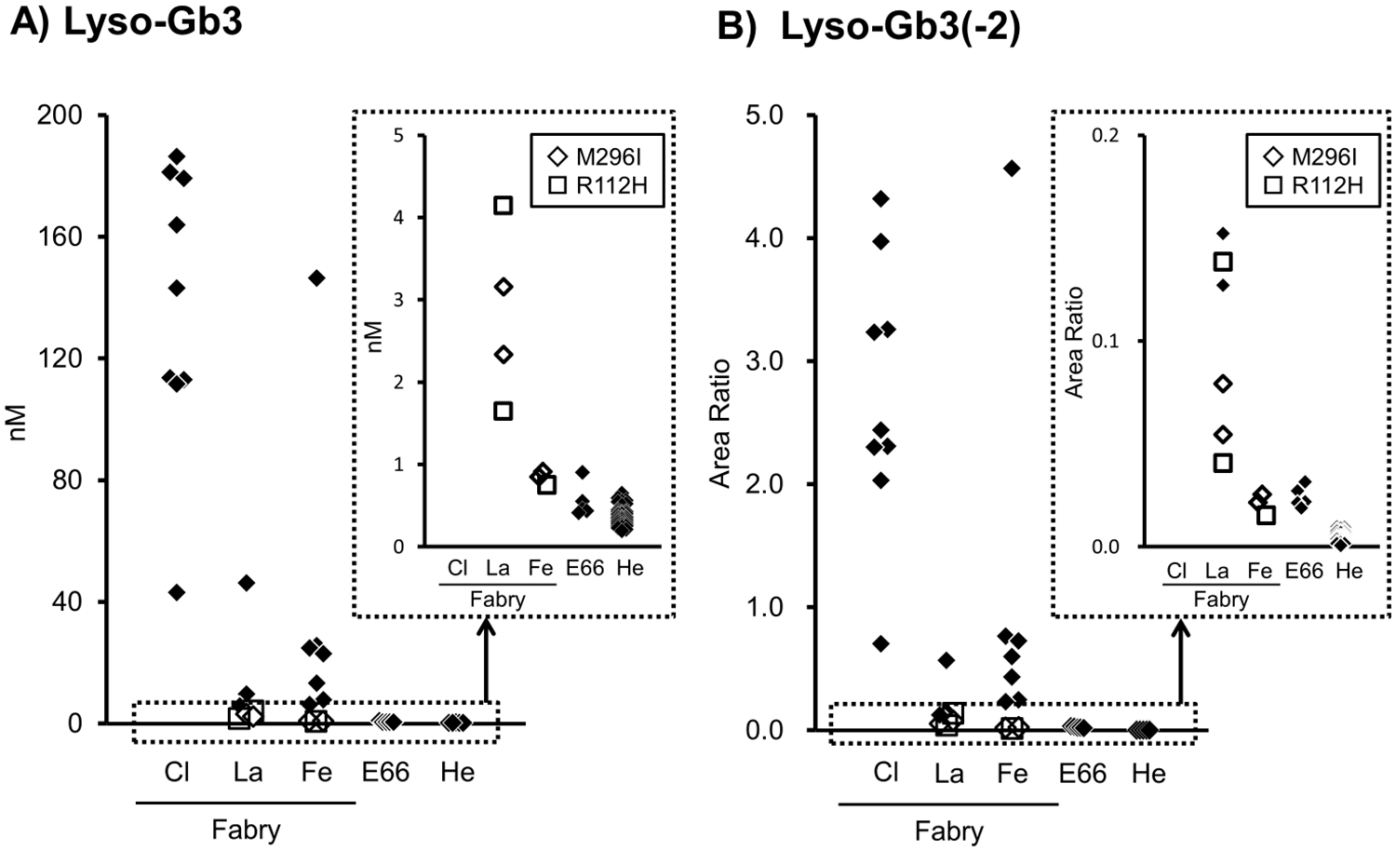
Plasma concentrations of A) lyso-Gb3 (nM) and B) lyso-Gb3(-2) (area ratio of lyso-Gb3-IS) in classic Fabry males (Cl), later-onset Fabry males (La), Fabry females (Fe), functional variants with E66Q (E66), and healthy subjects (He). Fabry patients harboring M296I (◇) and R112H (□). Other subjects (◆). The lower concentration areas for Lyso-Gb3 and Lyso-Gb3 (-2) are enlarged in the small right graphs.

Among the later-onset Fabry males, the lyso-Gb3 concentrations in the patients with R112H were 1.6 and 4.1 nM, and those in the patients with M296I 2.3 and 3.2 nM, all of which being lower than the concentrations in the other later-onset Fabry males, but higher than those in the functional variants and the healthy subjects (Table 1 and Fig 2A). Among the Fabry females, the lyso-Gb3 concentrations in the patients with R112H and M296I were 0.75, and 0.85 and 0.91 nM, respectively, being lower than those in the other Fabry females but slightly higher than those in the healthy subjects. However, it was difficult to determine the cut-off value for differentiating them from functional variants.

As references, the plasma lyso-Gb3 concentrations determined by the conventional HPLC method are summarized in Table 1. Although the plasma lyso-Gb3 concentrations determined by the nano-LC-MS/MS method tended to be higher than those obtained by the HPLC method, they were almost parallel with each other.

### Plasma concentrations of lyso-Gb3 analogues in Fabry patients, functional variants and healthy subjects

As mentioned above, eight lyso-Gb3 analogues were detected in plasma from the Fabry patients. Among the eight lyso-Gb3 analogues, two (lyso-Gb3(-2) of m/z 784 and lyso-Gb3(+34) of m/z 820) were detected in plasma from all the healthy subjects with our assay system. As to the functional variants, small amounts of lyso-Gb3(-2), lyso-Gb3(-28), lyso-Gb3(+16), lyso-Gb3(+18), lyso-Gb3(+34), and lyso-Gb3(+50) were detected, but not lyso-Gb3(-12) or lyso-Gb3(+14). On the other hand, Lyso-Gb3(-12) and lyso-Gb3(+14), which had been detected in urine but not in plasma in a previous study [16–18], were successfully detected in plasma from the Fabry patients by means of the nano-LC-MS/MS method. The plasma concentrations of the eight lyso-Gb3 analogues in the Fabry subgroups were increased compared with those in the functional variants and healthy subjects, and lyso-Gb3(-2) was the most abundant among the eight analogues (S2 Fig).

In the later-onset Fabry males, the area ratios of lyso-Gb3(-2) in the patients with R112H were 0.041 and 0.14, and those in the patients with M296I 0.055 and 0.079, all of which were higher than those in the functional variants and the healthy subjects (Fig 2B). In the Fabry females, the area ratios of lyso-Gb3(-2) in the patients with R112H and M296I were 0.015, and 0.021 and 0.025, respectively, being lower than those in the Fabry females with the other mutations. There was no difference in the plasma lyso-Gb3(-2) concentration between the female patients with R112H and M296I, and the functional variants.

## Discussion

We have developed a new sensitive method for measuring plasma lyso-Gb3 involving nano-LC-MS/MS. Up to now, quantification of lyso-Gb3 by only HPLC [7–10,12] and mass spectrometry [11,13–17] has been reported. The LOD of lyso-Gb3 with our assay method is 0.01 nM, which is lower than that (2–10 nM) for HPLC and that (0.05 nM) for the most sensitive assay method involving mass spectrometry (UPLC-MS/MS) [14]. Overall, our method is capable of sensitive quantification of lyso-Gb3 and its analogues.

The measurement of plasma lyso-Gb3 by means of this new method revealed that the plasma lyso-Gb3 concentrations in all the subgroups of Fabry patients (classic Fabry males, later-onset Fabry males and Fabry females) were higher than those in the healthy subjects and the functional variants with E66Q, which has been frequently found in the Japanese and Korean populations [26,27]. Furthermore, the plasma lyso-Gb3 concentrations tended to be high in Fabry patients whose disease stage had proceeded, i.e., in those who had developed heart disease (Table 1). These results show that plasma lyso-Gb3 is a useful biomarker of Fabry disease.

R112H is a missense mutation leading to the substitution of histidine for arginine112. Due to residual GLA enzyme activity, patients with this mutation exhibit the later-onset phenotype [7,8,28]. M296I is a missense mutation leading to the substitution of isoleucine for methionine296, which is common in Japanese Fabry patients exhibiting the later-onset phenotype. Patients with this mutation have residual GLA activity, and usually manifest no childhood symptoms, such as acroparesthesia, angiokeratomas, hypohidrosis, or corneal opacities, but develop renal and/or cardiac disease [12,29]. Previous examinations revealed that the plasma lyso-Gb3 concentrations in male patients with R112H and M296I were below or near the limit of determination [7,8,12,30]. However, our new sensitive method allowed successful determination of the lyso-Gb3 concentrations in such patients. Analysis involving nano-LC-MS/MS may be useful for addressing such Fabry patients, whose plasma lyso-Gb3 concentrations are low, as a tool to evaluate the response to ERT or other therapies.

The analysis with the new assay technique revealed that, regardless of the type of mutation, the plasma lyso-Gb3 concentrations in the male patients were higher than those in the functional variants and healthy subjects. On the other hand, as the Fabry females with R112H or M296I exhibited plasma lyso-Gb3 concentrations near those of the functional variants and the healthy subjects, it seems to be difficult to set a clear cut-off value for diagnosing Fabry females. Recently, Niemann et al. reported that Fabry patients with atypical mutations had lower lyso-Gb3 concentrations than those with classic Fabry disease, and that a cut-off value of 2.7 ng/mL (corresponding to 3.4 nM) of lyso-Gb3 in plasma separated the two groups [31]. However, our results suggest that there is overlap between the two groups, although our assay method is different from that of Niemann’s group.

Eight lyso-Gb3-related analogues were detected in plasma from Fabry patients with our method. This is the first report of lyso-Gb3(-12) and lyso-Gb3(+14) being detected in plasma from Fabry patients. Most of the Fabry patients exhibited higher plasma concentrations of the lyso-Gb3 analogues, especially of lyso-Gb3(-2), than those in the healthy subjects. This finding suggests that not only lyso-Gb3 but also lyso-Gb3(-2), another name for lyso-ene-Gb3, could be a useful biomarker for diagnosing Fabry patients, considering that lyso-Gb3 and its analogues may accumulate in the organs and tissues of Fabry patients according to their own metabolic pathways.

In conclusion, nano-LC-MS/MS could be used to determine the plasma concentrations of lyso-Gb3 and its analogues with high sensitivity, and revealed that they are useful biomarkers for Fabry disease.

## Supporting Information

S1 Fig MS chromatograms of lyso-Gb3, lyso-Gb3-IS and lyso-Gb3 analogues from a classical Fabry patient acquired by targeted MS/MS analysis. Triangles (▼) indicate the target peaks for quantification.
(TIF)

S2 Fig Plasma concentrations of lyso-Gb3 analogues (Area ratio of lyso-Gb3-IS) in classic Fabry males (Cl), later-onset Fabry males (La), Fabry females (Fe), functional variants with E66Q (E66), and healthy subjects (He). (TIF)

S1 Table Intra-day assaying of lyso-Gb3 in charcoal-treated plasma. (PDF)

S2 Table Inter-day assaying of lyso-Gb3 in charcoal-treated plasma. (PDF)

S3 Table Recovery of lyso-Gb3. (PDF)

S4 Table Precision for lyso-Gb3 analogues. (PDF)

S5 Table Stability of lyso-Gb3 and its two analogues. (PDF)

## References

[1] Desnick RJ, Ioannou YA, Eng CM. α-Galactosidase A deficiency: Fabry disease In: Scriver CR, Sly WA, Beaudet A L, Valle D, eds. The Metabolic and Molecular Bases of Inherited Disease, eighth ed. New York: McGraw-Hill 2001 pp. 3733–3774.

[2] Nance CS, Klein CJ, Banikazemi M, Dikman SH, Phelps RG, McArthur JC, et al Later-onset Fabry disease: an adult variant presenting with the cramp-fasciculation syndrome. Arch Neurol. 2006;63:453–457. 16533976 10.1001/archneur.63.3.453

[3] MacDermot KD, Holmes A, Miners AH. Anderson-Fabry disease: clinical manifestations and impact of disease in a cohort of 60 obligate carrier females. J Med Genet. 2001;38:769–775. 11732485 10.1136/jmg.38.11.769 PMC1734754

[4] Schiffmann R, Murray GJ, Treco D, Daniel P, Sellos-Moura M, Myers M, et al Infusion of alpha-galactosidase A reduces tissue globotriaosylceramide storage in patients with Fabry disease. Proc Natl Acad Sci U S A 2000;97:365–370. 10618424 10.1073/pnas.97.1.365 PMC26669

[5] Eng CM, Guffon N, Wilcox WR, Germain DP, Lee P, Waldek S, et al Safety and efficacy of recombinant human alpha-galactosidase A-replacement therapy in Fabry's disease. N Engl J Med. 2001;345: 9–16. 11439963 10.1056/NEJM200107053450102

[6] Young E, Mills K, Morris P, Vellodi A, Lee P, Waldek S, et al Is globotriaosylceramide a useful biomarker in Fabry disease? Acta Paediatr. 2005;94:51–54. 10.1111/j.1651-2227.2005.tb02112.x 15895713

[7] Aerts JM, Groener JE, Kuiper S, Donker-Koopman WE, Strijland A, Ottenhoff R, et al Elevated globotriaosylsphingosine is a hallmark of Fabry disease. Proc Natl Acad Sci U S A. 2008;105:2812–2817. 10.1073/pnas.0712309105 18287059 PMC2268542

[8] Rombach SM, Dekker N, Bouwman MG, Linthorst GE, Zwinderman AH, Wijburg FA, et al Plasma globotriaosylsphingosine: Diagnostic value and relation to clinical manifestations of Fabry disease. Biochim Biophys Acta. 2010;1802:741–748. 10.1016/j.bbadis.2010.05.003 20471476

[9] van Breemen MJ, Rombach SM, Dekker N, Poorthuis BJ, Linthorst GE, Zwinderman AH, et al Reduction of elevated plasma globotriaosylsphingosine in patients with classic Fabry disease following enzyme replacement therapy. Biochim Biophys Acta. 2011;1812:70–76. 10.1016/j.bbadis.2010.09.007 20851180

[10] Togawa T, Kodama T, Suzuki T, Sugawara K, Tsukimura T, Ohashi T, et al Plasma globotriaosylsphingosine as a biomarker of Fabry disease. Mol Genet Metab. 2010;100:257–261. 10.1016/j.ymgme.2010.03.020 20409739

[11] Lukas J, Giese AK, Markoff A, Grittner U, Kolodny E, Mascher H, et al Functional characterisation of alpha-galactosidase A mutations as a basis for a new classification system in Fabry disease. PLoS Genet. 2013;9:e1003632 10.1371/journal.pgen.1003632 23935525 PMC3731228

[12] Mitobe S, Togawa T, Tsukimura T, Kodama T, Tanaka T, Doi K, et al Mutant α-galactosidase A with M296I does not cause elevation of the plasma globotriaosylsphingosine level. Mol Genet Metab. 2012;107:623–626. 10.1016/j.ymgme.2012.07.003 22841442

[13] Krüger R, Tholey A, Jakoby T, Vogelsberger R, Mönnikes R, Rossmann H, et al Quantification of the Fabry marker lysoGb3 in human plasma by tandem mass spectrometry. J Chromatogr B Analyt Technol Biomed Life Sci. 2012;883–884:128–135. 10.1016/j.jchromb.2011.11.020 22138589

[14] Gold H, Mirzaian M, Dekker N, Joao Ferraz M, Lugtenburg J, Codée JD, et al Quantification of globotriaosylsphingosine in plasma and urine of Fabry patients by stable isotope ultraperformance liquid chromatography-tandem mass spectrometry. Clin Chem. 2013;59:547–556. 10.1373/clinchem.2012.192138 23237761

[15] Boutin M, Gagnon R, Lavoie P, Auray-Blais C. LC-MS/MS analysis of plasma lyso-Gb3 in Fabry disease. Clin Chim Acta. 2012;414:273–280. 10.1016/j.cca.2012.09.026 23041216

[16] Lavoie P, Boutin M, Auray-Blais C. Multiplex analysis of novel urinary lyso-Gb3-related biomarkers for Fabry disease by tandem mass spectrometry. Anal Chem. 2013;85:1743–1752. 10.1021/ac303033v 23248976

[17] Boutin M, Auray-Blais C. Multiplex tandem mass spectrometry analysis of novel plasma lyso-Gb3-related analogues in Fabry disease. Anal Chem. 2014;86:3476–3483 10.1021/ac404000d 24634980

[18] Auray-Blais C, Boutin M, Gagnon R, Dupont FO, Lavoie P, Clarke JT. Urinary globotriaosylsphingosine-related biomarkers for Fabry disease targeted by metabolomics. Anal Chem. 2012;84:2745–2753. 10.1021/ac203433e 22309310

[19] Dupont FO, Gagnon R, Boutin M, Auray-Blais C. A metabolomic study reveals novel plasma lyso-Gb3 analogs as Fabry disease biomarkers. Curr Med Chem. 2013;20:280–288. 23092136 10.2174/092986713804806685

[20] Karson KE, Novotny M. Separation efficiency of slurry-packed liquid chromatography microcolumns with very small inner diameters. Anal Chem. 1988;60:1662–1665 3232810 10.1021/ac00168a006

[21] Ishihama Y. Proteomic LC-MS systems using nanoscale liquid chromatography with tandem mass spectrometry. J Chromatogr A. 2005;1067:73–83. 15844511 10.1016/j.chroma.2004.10.107

[22] Karu K, Turton J, Wang Y, Griffiths WJ. Nano-liquid chromatography-tandem mass spectrometry analysis of oxysterols in brain: monitoring of cholesterol autoxidation. Chem Phys Lipids. 2011;164:411–424. 10.1016/j.chemphyslip.2011.04.011 21575613

[23] Thomas D, Eberle M, Schiffmann S, Zhang DD, Geisslinger G, Ferreirós N. Nano-LC-MS/MS for the quantitation of ceramides in mice cerebrospinal fluid using minimal sample volume. Talanta. 2013;116:912–918. 10.1016/j.talanta.2013.07.057 24148494

[24] Fedorova G, Randak T, Lindberg RH, Grabic R. Comparison of the quantitative performance of a Q-Exactive high-resolution mass spectrometer with that of a triple quadrupole tandem mass spectrometer for the analysis of illicit drugs in wastewater. Rapid Commun Mass Spectrom. 2013;27:1751–1762. 10.1002/rcm.6628 23821568

[25] Gold H, Boot RG, Aerts JM, Overkleeft HS, Codée JD, van der Marel GA. A concise synthesis of globotriaosylsphingosine. Eur J Org Chem. 2011:1652–1663.

[26] Lee BH, Heo SH, Kim GH, Park JY, Kim WS, Kang DH, et al Mutations of the GLA gene in Korean patients with Fabry disease and frequency of the E66Q allele as a functional variant in Korean newborns. J Hum Genet. 2010;55:512–517. 10.1038/jhg.2010.58 20505683

[27] Togawa T, Tsukimura T, Kodama T, Tanaka T, Kawashima I, Saito S, et al Fabry disease: biochemical, pathological and structural studies of the α-galactosidase A with E66Q amino acid substitution. Mol Genet Metab. 2012;105:615–620. 10.1016/j.ymgme.2012.01.010 22305854

[28] Eng CM, Niehaus DJ, Enriquez AL, Burgert TS, Ludman MD, Desnick RJ. Fabry disease: twenty-three mutations including sense and antisense CpG alterations and identification of a deletional hot-spot in the alpha-galactosidase A gene. Hum Mol Genet. 1994;3:1795–1799. 7531540 10.1093/hmg/3.10.1795

[29] Nakao S, Takenaka T, Maeda M, Kodama C, Tanaka A, Tahara M, et al An atypical variant of Fabry's disease in men with left ventricular hypertrophy. N Engl J Med. 1995;333:288–293. 7596372 10.1056/NEJM199508033330504

[30] Tsukimura T, Nakano S, Togawa T, Tanaka T, Saito S, Ohno K, et al Plasma mutant α-galactosidase A protein and globotriaosylsphingosine level in Fabry disease. Mol Genet Metab Reports. 2014;1:288–298. 10.1016/j.ymgmr.2014.07.005 PMC5121323 27896103

[31] Niemann M, Rolfs A, Störk S, Bijnens B, Breunig F, Beer M, Ertl G, et al Gene mutations versus clinically relevant phenotypes: lyso-Gb3 defines Fabry disease. Circ Cardiovasc Genet. 2014;7:8–16. 10.1161/CIRCGENETICS.113.000249 24395922

