# Supplementary figures and images for: Nano-LC-MS/MS for Quantification of Lyso-Gb3 and Its Analogues Reveals a Useful Biomarker for Fabry Disease
Source: PLoS One. 2015 May 12;10(5):e0127048. doi: 10.1371/journal.pone.0127048 (PMC4428877; doi:10.1371/journal.pone.0127048)

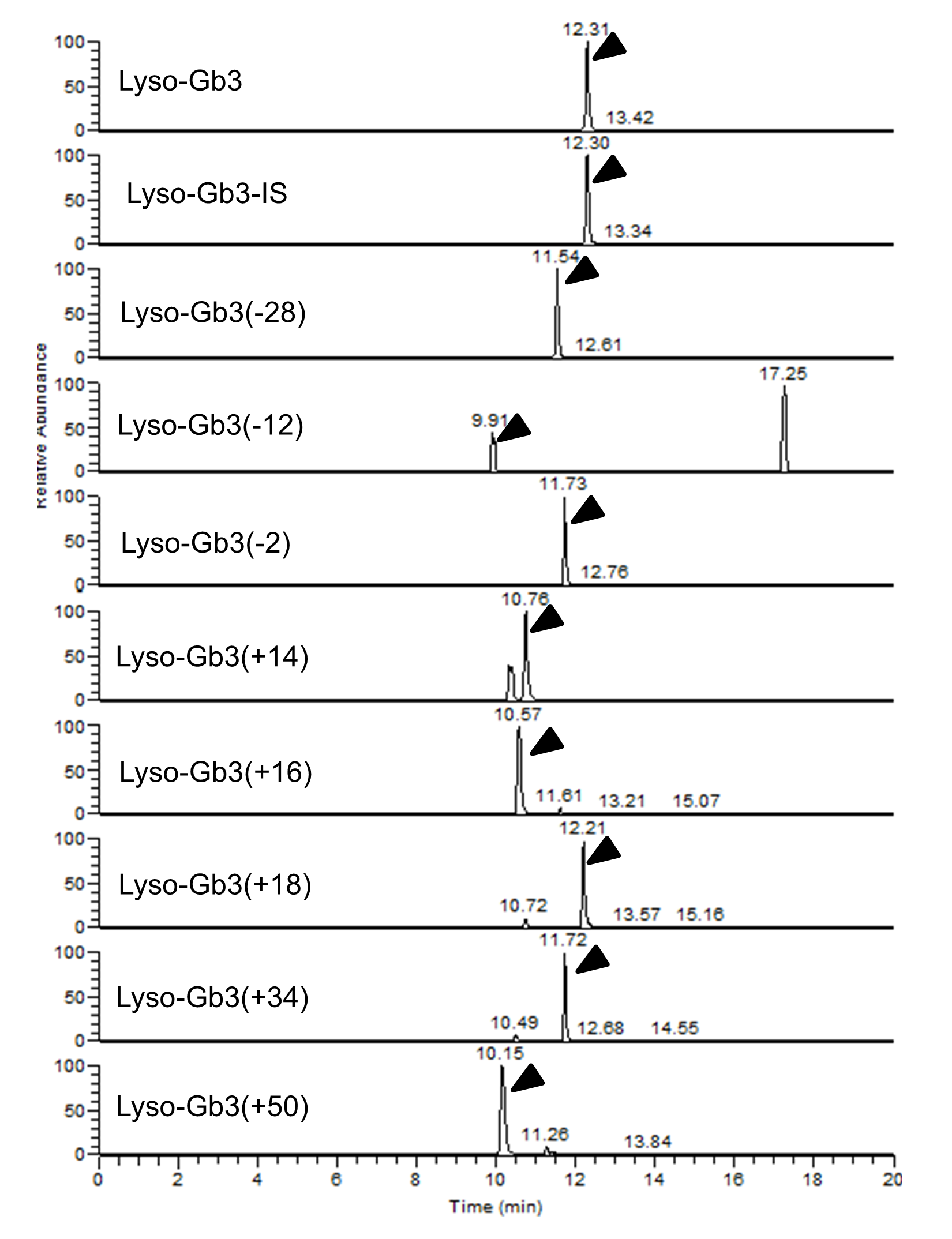

Supplement: S1 Fig — Triangles (▼) indicate the target peaks for quantification. (TIF) [file pone.0127048.s001.tif]

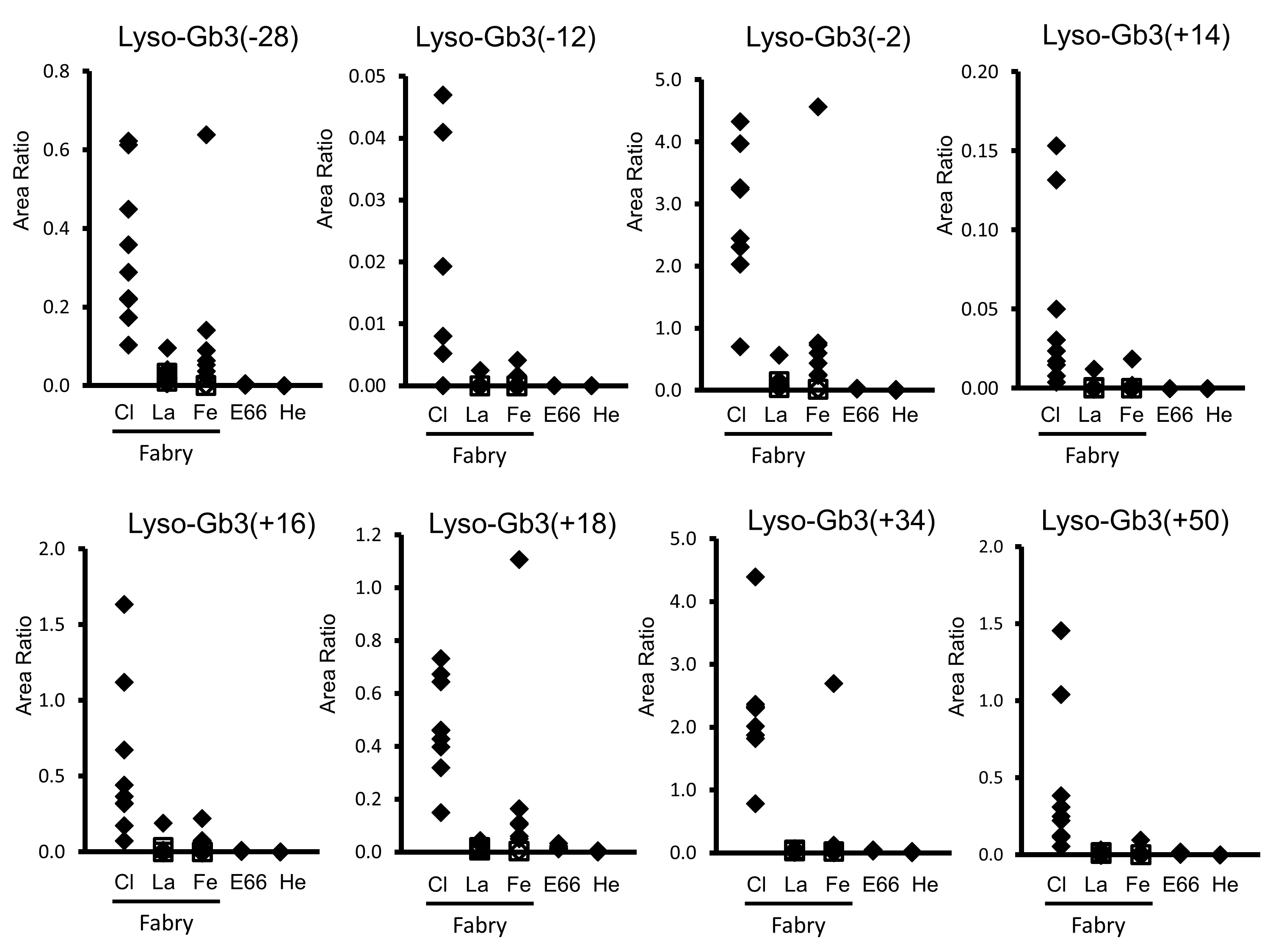

Supplement: S2 Fig — (TIF) [file pone.0127048.s002.tif]
