# Supplementary material for: Nano-LC-MS/MS for Quantification of Lyso-Gb3 and Its Analogues Reveals a Useful Biomarker for Fabry Disease
Source: PLoS One. 2015 May 12;10(5):e0127048. doi: 10.1371/journal.pone.0127048 (PMC4428877; doi:10.1371/journal.pone.0127048)
Supplement: S1 Table — (PDF) [file pone.0127048.s003.pdf]

Table S1. Intra-day assaying of lyso-Gb3 in charcoal-treated plasma.

Spiked concentration (nM)	Determined concentration (nM)	Mean determined concentration (nM)	S.D. (nM)	Precision (%)	Accuracy (%)
0.080	0.090<br>0.082<br>0.088<br>0.086<br>0.090	0.087	0.003	3.7	9.1
0.40	0.35<br>0.36<br>0.37<br>0.38<br>0.36	0.36	0.01	3.0	-9.7
10	9.0<br>8.7<br>9.3<br>9.1<br>9.2	9.1	0.2	2.4	-9.5
200	205<br>198<br>206<br>206<br>204	204	3	1.6	2.0
