# Supplementary material for: Nano-LC-MS/MS for Quantification of Lyso-Gb3 and Its Analogues Reveals a Useful Biomarker for Fabry Disease
Source: PLoS One. 2015 May 12;10(5):e0127048. doi: 10.1371/journal.pone.0127048 (PMC4428877; doi:10.1371/journal.pone.0127048)
Supplement: S2 Table — (PDF) [file pone.0127048.s004.pdf]

Table S2. Inter-day assaying of lyso-Gb3 in charcoal-treated plasma.

Spiked concentration (nM)	Day	Determined concentration (nM)	Mean determined concentration (nM)	S.D. (nM)	Precision (%)	Accuracy (%)	Overall (n=25)			
							Mean determined concentration (nM)	S.D. (nM)	Precision (%)	Accuracy (%)
	1	0.090<br>0.082<br>0.088<br>0.086<br>0.090	0.09	0.00	3.7	9.1				
	2	0.084<br>0.084<br>0.081<br>0.074<br>0.078	0.08	0.00	5.3	0.2				
0.08	3	0.074<br>0.066<br>0.071<br>0.063<br>0.075	0.07	0.01	7.5	-12.8	0.08	0.01	18.4	-3.1
	4	0.059<br>0.078<br>0.056<br>0.054<br>0.060	0.06	0.01	15.5	-23.4				
	5	0.076<br>0.087<br>0.085<br>0.122<br>0.074	0.09	0.02	21.7	11.2				
	1	0.35<br>0.36<br>0.37<br>0.38<br>0.36	0.36	0.01	3.0	-9.7				
	2	0.35<br>0.37<br>0.33<br>0.35<br>0.35	0.35	0.01	3.5	-12.8				
0.40	3	0.37<br>0.36<br>0.35<br>0.37<br>0.36	0.36	0.01	2.3	-9.7	0.35	0.02	4.7	-13.0
	4	0.33<br>0.33<br>0.33<br>0.31<br>0.33	0.33	0.01	3.2	-18.5				
	5	0.35<br>0.34<br>0.35<br>0.34<br>0.33	0.34	0.01	2.6	-14.4				

Spiked concentration (nM)	Day	Determined concentration (nM)	Mean determined concentration (nM)	S.D. (nM)	Precision (%)	Accuracy (%)	Overall (n=25)			
							Mean determined concentration (nM)	S.D. (nM)	Precision (%)	Accuracy (%)
	1	9.0<br>8.7<br>9.3<br>9.1<br>9.2	9.1	0.2	2.4	-9.5				
	2	9.2<br>9.2<br>9.3<br>9.1<br>9.2	9.2	0.0	0.4	-8.0				
10	3	9.4<br>9.5<br>9.0<br>9.0<br>10.0	9.3	0.4	4.6	-6.5	8.95	0.47	5.3	-10.5
	4	7.8<br>8.2<br>8.2<br>8.6<br>8.5	8.3	0.3	3.7	-17.3				
	5	9.1<br>9.0<br>8.8<br>9.3<br>8.3	8.9	0.4	4.2	-11.1				
	1	205<br>198<br>206<br>206<br>204	204	3	1.6	2.0				
	2	201<br>206<br>203<br>203<br>209	204	3	1.6	2.2				
200	3	220<br>209<br>201<br>199<br>187	203	12	6.0	1.6	204	5.96	2.9	2.1
	4	197<br>201<br>201<br>205<br>204	202	3	1.6	0.8				
	5	204<br>210<br>209<br>208<br>209	208	2	1.2	3.9				
