# Supplementary material for: Nano-LC-MS/MS for Quantification of Lyso-Gb3 and Its Analogues Reveals a Useful Biomarker for Fabry Disease
Source: PLoS One. 2015 May 12;10(5):e0127048. doi: 10.1371/journal.pone.0127048 (PMC4428877; doi:10.1371/journal.pone.0127048)
Supplement: S3 Table — (PDF) [file pone.0127048.s005.pdf]

Table S3. Recovery of lyso-Gb3.

Sample name		Peak area: Lyso-Gb3	Peak area: Lyso-Gb3-IS	Peak area ratio: Lyso-Gb3 / Lyso-Gb3-IS	Mean peak area ratio	Recovery (%)
QCL	Recovery	222233	21568203	0.010	0.012	50
		265358	20407486	0.013		
		228035	20454159	0.011		
		251204	19626267	0.013		
		256904	20162160	0.013		
	Control	597845	25631491	0.023	0.024	-
		556428	22995468	0.024		
		560318	23117647	0.024		
		564116	22744450	0.025		
		537043	23022897	0.023		
QCM	Recovery	8455225	24957551	0.34	0.33	54
		8413904	24983537	0.34		
		7999205	23774904	0.34		
		8117852	24656760	0.33		
		7400674	22972562	0.32		
	Control	15337801	24880672	0.62	0.61	-
		14988083	23164373	0.65		
		14744547	24478187	0.60		
		15700478	26258309	0.60		
		15427579	25783518	0.60		
QCH	Recovery	216284989	30742564	7.0	7.3	50
		206298651	28135604	7.3		
		202578296	28455672	7.1		
		217300438	28330117	7.7		
		204571374	28817681	7.1		
	Control	406887118	27306774	15	14	-
		378599731	27722681	14		
		392977978	27547315	14		
		370282945	25365325	15		
		346756729	23593515	15		
