# Supplementary material for: Nano-LC-MS/MS for Quantification of Lyso-Gb3 and Its Analogues Reveals a Useful Biomarker for Fabry Disease
Source: PLoS One. 2015 May 12;10(5):e0127048. doi: 10.1371/journal.pone.0127048 (PMC4428877; doi:10.1371/journal.pone.0127048)
Supplement: S4 Table — (PDF) [file pone.0127048.s006.pdf]

Table S4. Precision for Lyso-Gb3 analogues.

Sample name	Lyso-Gb3(-28)				Lyso-Gb3(-12)				Lyso-Gb3(-2)				Lyso-Gb3(+14)			
	Determined concentration (Area ratio)	Mean determined concentration (Area ratio)	S.D. (Area ratio)	Precision (%)	Determined concentration (Area ratio)	Mean determined concentration (Area ratio)	S.D. (Area ratio)	Precision (%)	Determined concentration (Area ratio)	Mean determined concentration (Area ratio)	S.D. (Area ratio)	Precision (%)	Determined concentration (Area ratio)	Mean determined concentration (Area ratio)	S.D. (Area ratio)	Precision (%)
	N.D				N.D				0.012				N.D			
	N.D				N.D				0.012				N.D			
Low	N.D				N.D				0.012	0.012	0.001	5.4	N.D			
	N.D				N.D				0.011				N.D			
	N.D				N.D				0.011				N.D			
	0.019				N.D				0.15				0.00089			
	0.022				N.D				0.15				0.0015			
Middle	0.017	0.019	0.002	9.0	N.D				0.15	0.15	0.01	5.5	0.0016	0.0015	0.0005	>20
	0.018				N.D				0.14				0.0013			
	0.019				N.D				0.13				0.0023			
	0.38				0.022				2.6				0.093			
	0.44				0.024				2.8				0.12			
High	0.43	0.43	0.03	6.5	0.027	0.024	0.002	8.2	3.2	2.8	0.2	7.9	0.11	0.11	0.01	9.3
	0.45				0.027				2.7				0.11			
	0.44				0.023				2.8				0.10			

Sample name	Lyso-Gb3(+16)				Lyso-Gb3(+18)				Lyso-Gb3(+34)				Lyso-Gb3(+50)			
	Determined concentration (Area ratio)	Mean determined concentration (Area ratio)	S.D. (Area ratio)	Precision (%)	Determined concentration (Area ratio)	Mean determined concentration (Area ratio)	S.D. (Area ratio)	Precision (%)	Determined concentration (Area ratio)	Mean determined concentration (Area ratio)	S.D. (Area ratio)	Precision (%)	Determined concentration (Area ratio)	Mean determined concentration (Area ratio)	S.D. (Area ratio)	Precision (%)
	0.00019				0.0085				0.019				0.00045			
	N.D				0.0086				0.019				0.00062			
Low	0.00050	0.00014	0.00022	>20	0.0067	0.0073	0.0012	17	0.023	0.020	0.002	9.2	0.00075	0.00049	0.00020	>20
	N.D				0.0072				0.018				0.00039			
	N.D				0.0057				0.020				0.00024			
	0.030				0.037				0.15				0.034			
	0.028				0.045				0.14				0.031			
Middle	0.028	0.030	0.003	8.8	0.041	0.041	0.003	7.5	0.16	0.15	0.01	6.1	0.032	0.033	0.001	4.4
	0.034				0.039				0.14				0.032			
	0.032				0.041				0.15				0.034			
	0.46				0.82				2.8				0.62			
	0.56				0.76				2.7				0.80			
High	0.68	0.58	0.08	14.4	0.63	0.71	0.08	11.0	3.1	2.8	0.2	8.5	0.76	0.73	0.07	9.4
	0.58				0.67				2.5				0.74			
	0.64				0.68				2.8				0.72			

N.D: Not detected.
