# Supplementary material for: Nano-LC-MS/MS for Quantification of Lyso-Gb3 and Its Analogues Reveals a Useful Biomarker for Fabry Disease
Source: PLoS One. 2015 May 12;10(5):e0127048. doi: 10.1371/journal.pone.0127048 (PMC4428877; doi:10.1371/journal.pone.0127048)
Supplement: S5 Table — (PDF) [file pone.0127048.s007.pdf]

Table S5. Stability of lyso-Gb3 and its two analogues.

Condition	Time	Lyso-Gb3 (% Bias)	Lyso-Gb3(-12) (% Bias)	Lyso-Gb3(+14) (% Bias)
Three Freeze/Thaw Cycles	-	-1.8%	8.2%	3.0%
-20°C	2 Weeks	1.7%	-9.5%	-5.1%
4°C	3 hr	-0.2%	15.3%	-3.1%
	6 hr	1.2%	25.7%	26.5%
Room Temperature	3 hr	0.0%	36.3%	24.6%
	6 hr	1.5%	32.8%	11.6%
Autosampler	20 hr	1.4%	-5.3%	-11.1%
	30 hr	1.9%	-3.3%	0.9%
